# Potential Impact of MicroRNA Gene Polymorphisms in the Pathogenesis of Diabetes and Atherosclerotic Cardiovascular Disease

**DOI:** 10.3390/jpm9040051

**Published:** 2019-11-25

**Authors:** Imadeldin Elfaki, Rashid Mir, Mohammad Muzaffar Mir, Faisel M AbuDuhier, Abdullatif Taha Babakr, Jameel Barnawi

**Affiliations:** 1 Department of Biochemistry, Faculty of Science, University of Tabuk, Tabuk 71491, Saudi Arabia; 2 Department of Medical Lab Technology, Prince Fahd Bin Sultan Research chair, Faculty of Applied Medical Sciences, University of Tabuk, Tabuk 71491, Saudi Arabia; rashid@ut.edu.sa (R.M.); fabu-duhier@ut.edu.sa (F.M.A.); jbarnawi@ut.edu.sa (J.B.); 3 Department of Basic Medical Sciences, College of Medicine, University of Bisha, Bisha 61992, Saudi Arabia; mirmuzaffar11@gmail.com; 4 Department of Medical Biochemistry, Faculty of Medicine, Umm Al-Qura University, Makkah 57039, Saudi Arabia; abdullatiftaha@hotmail.com

**Keywords:** diabetes mellitus, cardiovascular disease, atherosclerosis, MicroRNA, single nucleotide polymorphisms (snps)

## Abstract

MicroRNAs (miRNAs) are endogenous, small (18–23 nucleotides), non-coding RNA molecules. They regulate the posttranscriptional expression of their target genes. MiRNAs control vital physiological processes such as metabolism, development, differentiation, cell cycle and apoptosis. The control of the gene expression by miRNAs requires efficient binding between the miRNA and their target mRNAs. Genome-wide association studies (GWASs) have suggested the association of single-nucleotide polymorphisms (SNPs) with certain diseases in various populations. Gene polymorphisms of miRNA target sites have been implicated in diseases such as cancers, diabetes, cardiovascular and Parkinson’s disease. Likewise, gene polymorphisms of miRNAs have been reported to be associated with diseases. In this review, we discuss the SNPs in miRNA genes that have been associated with diabetes and atherosclerotic cardiovascular disease in different populations. We also discuss briefly the potential underlining mechanisms through which these SNPs increase the risk of developing these diseases.

## 1. Introduction

**Figure 1:**
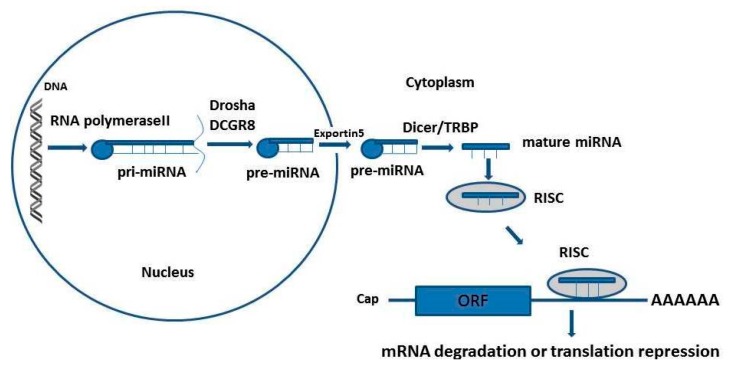
The canonical pathway of miRNA biogenesis in animal cells. This figure is modified from Yu et al., and Graves and Zeng, 2012 [3,8].

Micro RNAs (miRNAs) are short 22~24 nucleotides in length, single-stranded and non-coding RNA molecules that regulate post-transcriptional gene expression [1]. The discovery of the miRNAs has opened a new era in genome biology. They regulate gene expression by binding to the 3′ untranslated regions (3′UTRs) of their target mRNAs and thereby inhibit the translation [2]. There are three steps for canonical miRNA biogenesis in animals [3]. Firstly, it is transcribed by RNA polymerase II to a primary miRNA (pri-miRNA) that is about 100 nucleotides in length and has the structure of a stem-loop that fold back [4]. Secondly, in the nucleus, the pri-miRNA is processed into a precursor (pre-miRNA of about 60–70 nucleotides) by the nuclear RNase III Drosha and the RNA-binding protein DCGR8 [5]. Finally, the pre-miRNA is exported to the cytoplasm by exportin 5 where the mature 22- nucleotides miRNA is produced when pre-miRNA is cleaved by the cytoplasmic RNase III Dicer-TRBP (transactivation-response RNA binding protein) complexes [3,6,7,8]. The miRNAs inhibit or silence the mRNA translation by forming a ribonucleoprotein complex, called RNA-induced silencing complex (RISC) [9,10]. The mature miRNA then complexes with Argonaute proteins and form the RNA-induced silencing complex (RISC) [10]. In the non-canonical pathway, other enzymes than Drosha-DGCR8 and Dicer-TRBP are used as RNAses [3]. For instance, in the mirtron pathway, short hairpin introns are excised by splicing and lariat-debranching enzyme to generate intermediates of the RNA interference effectors or miRNAs [11]. MiRNAs can also be produced by other RNases from certain hairpin of non-coding species of RNA, for example, the transfer RNAs of small nucleolar RNAs [12,13]. The translation inhibition of the target mRNAs occurs by binding of the miRNA within the RISC to the complementary sequences in the 3′UTRs of target mRNAs (Figure 1) [10]. Then, there are two types of translation inhibition, the mRNA deadenylation and decay (degradation) or translation repression [9]. MicroRNAs are negative regulators of gene expression acting on the mRNA at the post-transcriptional levels [14]. They have been implicated in many various physiological processes such as development, metabolism, differentiation, proliferation, and responses to stress [9,15]. It has been estimated that more than 2000 miRNAs have been discovered, and these miRNAs regulate about 30% of human protein-coding genes [16,17]. A single gene may be regulated by many miRNAs. Whereas, a single miRNA may regulate many target genes [18]. Single-nucleotide polymorphisms (SNPs) are present in the genome at a frequency of 1 in 1kbp [19]. They are probably the cause of interindividual variations in a physiological trait such as curly hair [19], height and weight [20]. The SNPs can also be the cause of variation in drug response between individuals [21]. Moreover, they can be the cause of several pathophysiological disorders such as diabetes [22,23,24,25] cancers or atherosclerosis [21,26,27,28,29,30,31], obesity, hypertension, psychiatric disorders [20], inflammatory and autoimmune diseases [32]. SNPs at the miRNA gene may enhance, reduce expression, change maturation of miRNA, or disrupt miRNA-mRNA binding [33]. Disruption of miRNA-mRNA binding may contribute to the development of different types of human diseases. This disruption may result from gene polymorphism at the miRNA target site [34,35,36], or polymorphism in miRNA genes [27,29,30,37,38,39,40,41].

Some microRNAs are implicated in insulin secretion (such as miR-7 and miR-206) [42], and others in the development of pancreatic beta cells such as miR-375 and miR-124a [43,44]. Type 1 diabetes mellitus (T1DM) resulted from the destruction of pancreatic beta cells and lack of insulin [45]. Type 2 diabetes mellitus (T2DM) results from insulin resistance initiated in hepatocytes, skeletal muscles and adipose tissues [45]. Consequently, there is a higher demand for insulin, and beta cells then compensate by increasing insulin secretion until they get exhausted [46]. In fact, loss of function of beta cells (by insults such as inflammation and insulin resistance) is a basic cause of T1DM or T2DM [46].

MiRNAs play very important roles in glucose and fat metabolism. There is a different expression of some microRNAs between T2DM cases and controls in skeletal muscles such as miR-29 [47], in adipose tissues such as miR-21 [48], or in pancreatic islets such as MiR-375 [42].

Moreover, miRNAs have been implicated in key processes involved in the pathogenesis of cardiovascular disease (CVD) [10]. For example, they have been reported to be involved in cardiac development such as miR-208, miR-1, miR-133, miR-21 [49]. In addition, the cholesterol homeostasis has been reported to be regulated by the miR-10b, miR-33a/b, miR-106b, and others [10]. While, the miR-1, miR126-5p, miR-155, miR-218 and others involved in the regulation of endothelial cell homeostasis [10,50], and miR-9, miR-21, and others regulate the inflammatory response, for example, miR-21 and miR-26b) [10,51,52].

The roles of SNPs in microRNA target sites in disease pathogenesis have previously been reviewed [35,53]. In this review, we discuss the role of SNPs in pri-miRNA, pre-miRNA or mature miRNA sequences in the pathogenesis of diabetes and atherosclerosis-related cardiovascular disease.

## 2. Micro-RNA Gene Polymorphisms in Diabetes and Diabetes Complications

### 2.1. MiR-124a rs531564 G>C

The miR-124a is expressed in brain and pancreatic tissue [44]. The rs531564 (G Allele) of miR-124a has been associated with T2DM in Han Chinese and Italian populations [54,55]. MiR-124a is involved in pancreatic beta cells differentiation and proliferation as well as insulin secretion [44]. Moreover, the myotrophin gene is a target of miR-124a [44]. The myotrophin gene encodes a cytoplasmic protein that induces exocytosis and hormone secretion [44]. Moreover, miR-124a targets genes that are implicated in the function of the pancreatic beta cells such as *Mtpn*, *Foxa2*, *Flot2*, *Akt3*, *NeuroD1* and *Sirt1* [56]. Dysregulation of miR-124a would negatively affect insulin production and secretion [57]. It has also been reported that altered expression of miR-124a may lead to impaired beta cells function through miR-124a effects on its target genes that are involved in beta cell physiology [56].

### 2.2. MiR-375

MiR-375 is also expressed in the brain and pancreas and is regulated by transcription factors such as the neurogenic differentiation factor 1 and Pdx-1 which are both important for the development of pancreatic beta cells [44]. MiR-375 also regulates insulin secretion [43]. In addition, miR-375 has been shown to be significantly overexpressed in streptozotocin (STZ)-treated mice and non-obese diabetic mice before the onset of hyperglycemia [58]. Moreover, miR-375 has also significantly increased in STZ and cytokine-induced cell death in isolated mouse islets [58]. MiR-375 levels have decreased after the addition of an inhibitor of cell death [58]. Therefore, it is suggested that the circulating miR-375 levels can be used as a biomarker of beta cells destruction and a potential predictor of diabetes mellitus (DM) [58]. In fact, miR-375 has overlapping functions with miR-124a with respect to pancreatic beta cells development and insulin secretion [44]. However, to our knowledge, the role of miR-375 polymorphisms (e.g., rs1005317333, rs1003868351, rs1001341512, rs1003080648) in the induction of T2DM is yet to be elucidated.

### 2.3. MiR-146a rs2910164 C>G

**Figure 2:**
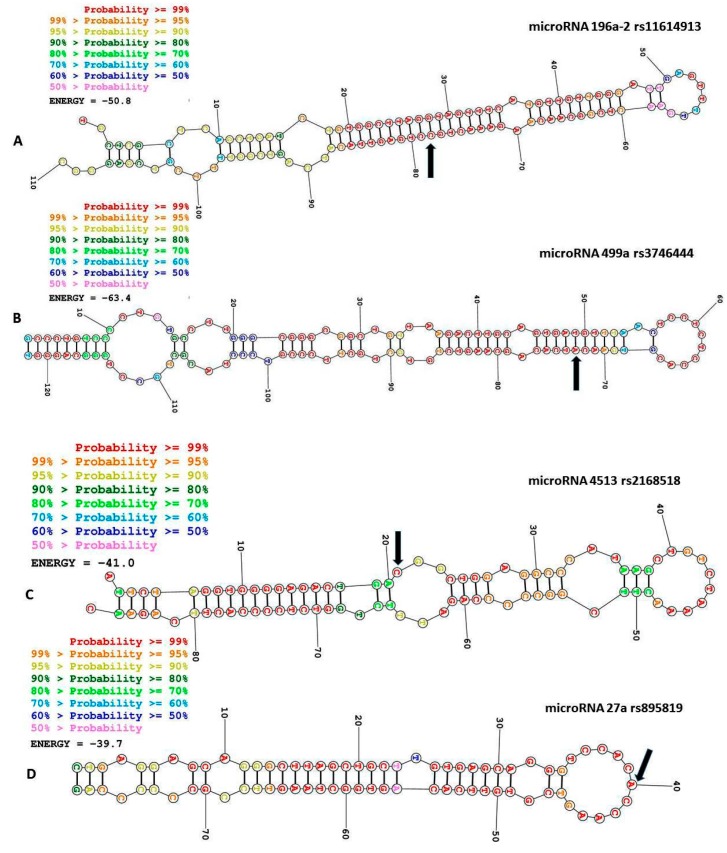

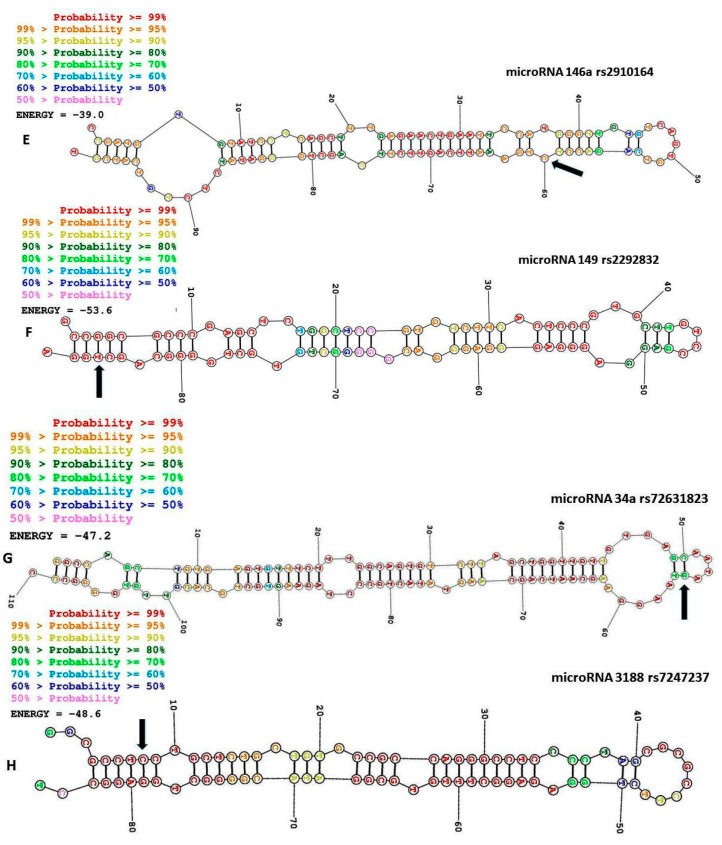
Structural predictions of the microRNAs. The sites of single-nucleotide polymorphisms (SNPs) in the predicted microRNA structures are indicated with arrows. (**A**) miR-196a2 (rs11614913) 78T>C, probably a stemp-loop SNP (**B**) miR-499 (rs3746444) 73A>G, probably a stemp-loop SNP (**C**) MIR-4513 (rs2168518) 21C>T probably a stemp-loop SNP (**D**) pre-miR-27a (rs895819) 40A>T, probably a stemp-loop SNP (**E**) miR-146a (rs2910164) 60G>C, probably a stemp-loop SNP (F) miR149 (rs2292832) 86T>C, probably a seed SNP (**G**) miR34a (rs72631823) 55G>A, probably a stemp-loop SNP (**H**) miR-3188 (rs7247237) 8C>T probably a seed SNP. The sites of the SNPs are indicated with arrows. This figure has been prepared using the webserver https://rna.urmc.rochester.edu/RNAstructureWeb/.

The rs2910164 has been reported to increase the risk to T2DM in Chinese population [54]. The rs2910164 have been reported to reduce the expression of pre- and mature miR-146a [59]. It has been shown that miR-146a attenuates the activity of NF-kappa B [60], and it has been reported that the activation of the NF-kappa B (and the inflammatory events in general) is an important factor in the pathophysiology and complications of diabetes [61,62,63]. In contrast to miR-34a (discussed below), increased levels of miR-146 do not influence the capacity of beta cells for insulin secretion, but rather increased beta cells’ apoptosis [64]. In a study conducted in the Caucasian population, Kaidonis et al., reported that rs2910164 is associated with diabetic nephropathy in T1DM patients and diabetic macular edema in T2DM patients [65]. The rs2910164 SNP is found within the seed sequence of the pre-miR-146a [66]. However, in our predicted structure, the rs2910164 SNP seems to be within the stem-loop of miR-146 (Figure 2E). It is suggested that the C allele of the rs2910164 would reduce the mature miR146a levels [65]. Reduced levels of mature miR-146a would lead to dysregulation of NF-kappa B-mediated inflammation which is implicated in the development of diabetes complications [65]. Ciccacci et al. also reported that the rs2910164 is associated with increased risk to diabetic polyneuropathy (DPN) in the Italian population [67], however, the C allele has a protective effect [67]. Furthermore, miR-146a rs2910164 has been reported to be associated with Preeclampsia in Gestational Diabetes in the Egyptian population [68]. It is suggested that the expression of miR-146a is correlated with the levels of plasma renalase enzyme that maintains renal blood pressure [68,69,70].

### 2.4. MiR-27a rs895819

The rs895819 has been reported to be associated with decreased risk to T2DM in Italian and Iranian populations [55,71]. It has been suggested that rs895819T>C increased the expression of the miR-27a [55,71,72,73]. It has been demonstrated that miR-27 exerts a negative effect on adipogenesis [74]. It has been suggested that miR-27a targets the PPAR gamma and hence negatively influence the differentiation of the adipocyte [75], and the down-regulation of miR-27a would result in dysregulation of adipose tissue and obesity [75]. The role of obesity in the induction of insulin resistance and T2DM is well known [76]. Therefore, wild miR-27a may have a protective role against obesity and hence T2DM. It is conceivable that more active miR-27a (with the CC genotype) has a more protective role against T2DM as reported in previous studies [55,71]. Nevertheless, Wang et al. 2015 have never observed the protecting effect of miR-27 against T2DM in the Chinese Han population [77]. Wang et al., 2015 suggested that this contradiction might be due to the different geographic locations, size of the samples, and ethnicity [77]. The G allele of miR-27a rs895819 was associated with increased risk for developing early cardiovascular autonomic neuropathy (CAN) in the Italian population [67].

### 2.5. MiR-34a rs72631823 G>A

The rs72631823 of mi-R34a has been reported to be associated with T2DM [78]. It is located within the terminal loop region of the pre-miRNA of miR-34a [78]. In an experiment conducted in 3 cell lines (β-cell line MIN6B1, pancreatic islets and Hela cells) transfected with the miR-34a, it has been shown that the expression of MIR34a has significantly increased with the A allele compared with the wild type (G allele). The increased expression of miR-34a bearing the A allele may be due to a more relaxed secondary structure of miR-34a with the A allele than that with the G allele [78], as the more relaxed micro-RNA secondary structure may be processed more sufficiently during biogenesis [78]. MIR34a has been reported to increase beta cells apoptosis and hence DM [78]. Dysfunction of pancreatic beta-cells that induced by the proinflammatory cytokines and palmitate increased the expression levels of miR-34a in β-cell line MIN6B1, pancreatic islets and islets from diabetic db/db mice [64,78]. Increased expression levels of miR-34a activate the p53 which leads to increased sensitivity to apoptosis and impaired nutrient-induced secretion of insulin [64]. The impaired insulin secretion is probably because of expression inhibition of the vesicle-associated membrane protein 2 (VAMP-2) [64]. VAMP-2 is a protein expressed in pancreatic beta cells and is critical for calcium-induced insulin exocytosis [79,80]. Inhibiting the expression of miR-34a resulted in decreased beta cells apoptosis [64,78,81]. It may be concluded that certain levels of miR-34a expression should be maintained within the pancreatic tissues.

### 2.6. MicroRNA Let-7a-2 (MIRLET-7A2 rs1143770)

The rs1143770 of Let-7 a-2 has been associated with diabetic nephropathy (DN) in the Chinese Han population [82]. Let-7 regulates several aspects of the metabolism of glucose [45]. It has been reported that knockdown of the Let-7 family with an antimiR can be used in anti-diabetic treatment [45]. Overexpression of Let-7 in all organs and in the pancreas, in particular, resulted in impaired glucose tolerance and decreased pancreatic insulin secretion induced by glucose [45]. In addition, knockdown of the Let-7 family with an antimiR treatment resulted in improvement of sensitivity to insulin in muscle cells and hepatocytes of obese mice [45], this treatment resulted in reduced fat mass in muscles as well as in hepatocytes [45]. The rs1143770 C>T in the regulatory region of Let-7 a-2 (CT/TT genotypes) is associated with increased risk for diabetes and diabetic nephropathy (DN) in the Chinese Han population [82]. It has been suggested that this polymorphism rs1143770 C>T causes a partial loss of cyclic adenosine monophosphate response element-binding protein (CREB) [82]. The CREB is a transcription factor implicated in DN [82,83].

### 2.7. MiR-155 rs767649 T>A

The TT genotype of rs767649 has been associated with protection against T1DM in the Brazilian population [66]. MiR-155 is overexpressed in immune cells (T, B lymphocytes, macrophages and dendritic cells) [66]. It has been reported that miR-155 is an immune response molecule [84]. For example, it is induced by bacterial lipopolysaccharide (LPS), Interferons-β, polyriboinosinic–polyribocytidylic acid (poly IC) or Tumor Necrosis Factor-α (TNF-α) [84]. It has been reported that MIR155 is a transactivational target of nuclear factor kappa B (NF-kappa B) [85], the NF-kappa B upregulates miR-155 [85]. The upregulated miR-155 then, through its targets IKKβ and IKKε, leads to the limitation of NF-kappa B [85]. It has been reported that when beta cells exposed to Interleukin-1 (IL-1), the NF-kappa B is activated and translocate to the nucleus. This activation modifies the expression of many genes that cause beta cells’ dysfunction and apoptosis [61]. It has been suggested that the A allele of the rs767649 is associated with reduced expression of miR-155 [66]. The reduced expressed miR-155 (with the A allele) would have inefficient control on activated NF-kappa B which predispose to T1DM [66].

### 2.8. MiR128a rs11888095 C>T

The rs11888095 has been reported to be associated with the development of diabetic polyneuropathy (DPN) in an Italian cohort [67]. Using computational analysis, it is suggested that miR128a targets include genes related to T2DM such as insulin receptor substrate 1 (IRS-1), calpain 10 (CAPN10), and peroxisome proliferator-activated receptor gamma (PPAR gamma) [67]. For example, IRS-1 is an important element in insulin signaling pathway and disturbance of IRS1 complexes may lead to insulin resistance and T2DM [86], while the PPAR gamma is involved in the pathogenesis of various diseases including T2DM [87].

### 2.9. MiR-3188 rs7247237 C>T

The rs7247237 has been reported to be associated with the incidence of vascular complications in T2DM patients from the Chinese population [88]. The rs7247237 SNP (Figure 2H) reduces the expression of mature MiR-3188 [88]. It is suggested that the decreased expression of mature MiR-3188 leads to overexpression of the glutathione S-transferase M1 (GSTM1) and Tribbles pseudokinase 3 (Trib3) [88]. The overexpression of GSTM1 and Trib3 results in reduced Nitric Oxide (NO) production in the endothelial cells through inhibition of endothelial NO synthase [88]. In T2DM, the endothelial dysfunction is caused by reduced bioavailability of endothelial NO synthase [89]. MiR-3188 has been reported to have a role in the regulation of mTOR and PI3K/Akt signaling pathway [90]. Therefore, the MiR-3188 rs7247237 polymorphism (resulting in reduced MiR-3188 expression) may also be involved in the induction of T2DM because impairment of PI3K/AKT signaling pathway results in insulin resistance and T2DM [91].

### 2.10. MiR-126 rs4636297 A>G

The rs4636297 (A-allele) has been reported to be associated with sight threatening diabetic retinopathy (DR) in an Australian cohort with T2DM [92]. It has been reported that miR-126 regulates the amount of circulating vascular endothelial growth factor (VEGF) [92,93]. VEGF is very important for the development and maintenance of the blood and lymphatic vessels [94]. MiR-126 suppresses the inhibitors of the VEGF pathway such as the phosphoinositol-3 kinase regulatory subunit 2 and the Sprouty-related protein SPRED1 [94]. The miR-126 rs4636297 polymorphism has been implicated in the development of ischemic stroke risk in the Chinese population [95]. Thus, miR-126 maintains angiogenesis and integrity of the vascular system [96]. Future research is required to obtain insight into the role of miR-126 rs4636297 polymorphism in the pathogenesis of other diabetic microvascular complications (Table 1).

## 3. Micro-RNA Gene Polymorphisms in Atherosclerotic Cardiovascular Disease

### 3.1. The miR-196a2 rs11614913 T>C

The rs11614913 of miR-196a2 (TT genotype) has been shown to be associated with increased risk to CVD in T2DM patients from the Polish population [37]. Recently, Fragoso et al., suggested that the rs11614913 is associated with coronary artery disease (CAD) in the Mexican population [97]. The rs11614913 (Figure 2A) polymorphism of miR-196a2 affects the expression of mature miR-196a2 and its binding to the target mRNAs [98]. The effect of rs11614913 on miR-196a2 may have an impact on the risk and progression of diseases [99,100]. This polymorphism affects the miR-196a-HOXB8-Shh signaling pathway, which is critical for cardiac septation, morphogenesis of the outflow tract and valve development [37,49]. Moreover, it has been reported that annexin A1 is regulated by miR-196a [101]. Annexin A1 was reported to attenuate cardiovascular complications in T1DM [102] and plays a protective role against the inflammatory process in atherosclerosis, myocardial infarction and stroke [103]. This protective role may be because annexin A1 reduces TNF-alpha levels [37]. The role of TNF-alpha and inflammatory cytokines in the induction of the inflammatory process (e.g., atherosclerosis) is well established [104]. The miR-196a2 (rs11614913) polymorphism may thus reduce the regulatory capacity of the annexin A1 by miR-196a [98,100].

### 3.2. MiR-499 rs3746444 A>G

The rs3746444 (GG genotype) which is present in 20 intron of cardiac β-myosin heavy chain 7B gene of human has been associated with myocardial infarction in Chinese and Egyptian populations [105,106,107]. MiR-499 (Figure 2B) is present in skeletal muscles, the myocardium and brain [105,108]. MiR-499 enhances the differentiation of myocardial cells [109]. Furthermore, miR-499 has been demonstrated to inhibit the pathway of mitochondrial apoptosis and protects the cardiac cells from injury induced by reactive oxygen species (ROS) [110]. Moreover, it has been reported that miR-499 promotes the recovery of the heart after myocardial infarction (MI) by enhancing the maturation of human cardiac stem cells [111]. Furthermore, targets of miR-499 include the interleukin proteins that are involved in the activation of inflammatory processes [112]. It has been also been reported that both isoforms of the catalytic subunit of calcineurin are targets of MIR499 [113]. Moreover, miR-499 decreases the apoptosis in cardiomyocytes by inhibition of dephosphorylation of dynamin-related protein-1 (Drp1) mediated by its direct target calcineurin [113]. Hence, miR-499 reduces the activation of the mitochondrial fission program mediated by Drp1 [113]. The mitochondrial fission is involved in the early stage of apoptosis [114]. Li et al. 2015 [105] concluded that the rs3746444 polymorphism induces CAD risk in 2 ways. Firstly, it disturbs miR-499 regulation of blood pressure; secondly, it hampers miR-499 anti-apoptotic effect in cardiomyocytes [105]. In a recent study by Li et al., it has been reported that the G allele of the rs3746444 is associated with ischemic stroke in the Chinese population [115]. Li et al., suggested that the association of rs3746444 with ischemic stroke because miR-499 has roles in the reduction of the activation of the mitochondrial fission program mediated by Drp1 [115], as well as it has roles in the regulation of expression of inflammatory cytokines such as C-reactive protein and interleukins [115]. The role of inflammatory cytokines in cardiovascular disease is well established [116]. MiR-499 polymorphism (rs3746444) has also been reported to be associated with cancers [117] and autoimmune diseases [118].

### 3.3. MiR-4513 rs2168518 C>T

The rs2168518 (TT genotype) was also reported to be associated with CAD in Chinese and south Indian populations [27,105]. The rs2168518 mutation (Figure 2C) decreases Mir-4513 activity [119]. The rs2168518 is associated with blood pressure, total lipids, total cholesterol, low-density lipoprotein cholesterol (LDL-C) and blood glucose [105,119]. Targets of miR-4513 include *PCSK1, BNC2, MTMR3, ANK3*, and *GOSR2* [119]. The rs2168518 polymorphism may affect the glucose and lipid homeostasis through regulation of these its targets [119]. *PCSK1* genetic variation has been associated with insulin sensitivity and obesity [120]. The *BNC2* gene is associated with HbA1c and type1 diabetes and is involved in the regulation of cholesterol metabolism [119]. The *MTMR3* gene is involved in LDL-C metabolism, while the *ANK3* gene is associated with higher systolic blood pressure [119]. *GOSR2* is associated with hypertension, CAD and myocardial infarction (MI) [119,121,122].

### 3.4. The Pre-MiR-27a rs895819 A>T

The rs895819 (GG genotype) has been associated with myocardial infarction in the Chinese Han population [5]. It has been reported that the GG genotype of rs895819 polymorphism (Figure 2D) is associated with reduced high-density lipoprotein cholesterol (HDL-C) levels, while the AG and AA genotypes are associated with elevated HDL-C levels [5]. MiR-27a was reported to regulate lipid metabolism by inhibition of several lipid metabolic genes such as fatty acid synthase (FASN), sterol regulatory element-binding proteins 1 and 2 (SREBP−1,2), peroxisome proliferator-activated receptor alpha and gamma (PPARα and γ), and apolipoprotein A1, B100 and E3 [123]. Moreover, MIR-27 also regulates lipid metabolism by decreasing lipogenesis and increasing the cellular secretion of lipids [123].

### 3.5. MiR-146a rs2910164 G>C

It was reported that the rs2910164 was associated with the risk to acute coronary syndrome (ACS) and CAD in Chinese, South African Indian and Korean populations respectively [100,124,125,126]. MIR146a polymorphism has been associated with total cholesterol, total lipids and C- reactive protein levels [124]. Moreover, it has been reported that miR-146a (with miR-155) regulates the pro-inflammatory NF-kappa B [59,60], and represses the MAP kinase pathway [127]. Furthermore, MIR146a was reported to be an important repressor of the inflammatory process [128]. It negatively regulates the production of proinflammatory cytokines TNF-α, IL-1β and chemokines IL-8, and RANTES in macrophages induced by vesicular stomatitis virus [129]. The C allele of (rs2910164G>C) reduces the expression of MIR146a [59], and decreases its anti-inflammation effects which may reduce the incidence of CAD. CAD is considered as a chronic inflammatory disease [130]. Jazdzewski et al., reported that in the C allele of the rs2910164G>C, the expression of pre- and mature miR-146a would be less by up to 1.8-fold from C allele compared with the G allele [59]. Jazdzewski et al., suggested that rs2910164G>C reduced expression of pre- and mature miR-146a [59]. The miR-146a is a NF-kappa B-dependent gene [59], and the role a NF-kappa B in atherosclerosis is well established [131]. It has been suggested that MIR146 decreased the activity of nuclear factor Kappa B in the toll-like receptor pathway by targeting the interleukin-1 receptor-associated kinase-1 (IRAK1) and TNF receptor associated factor-6 (TRAF6) [68]. The rs2910164 leads to reduced mature miR-146a which would increase the TNF-α, IRAK1, and TRAF6 which may increase atherosclerotic related inflammatory response [132].

### 3.6. MiR-149 rs2292832 T>C

The rs2292832 has been reported to be associated with CAD in Korean and Iranian populations [126,133]. This polymorphism is found in the precursor miR-149 and negatively affects its expression to mature miR-149 [126]. MiR-149 was reported to prevent mitochondrial related apoptosis through targeting the pro-apoptotic protein p53 upregulated modulator of apoptosis (Puma) [134]. Furthermore, knockdown of MIR149 renders the cardiomyocytes more sensitive to apoptotic stimulation, while MIR149 enforced expression enhances survival of cardiomyocytes [134]. In addition, miR-149 has been shown to be down-regulated in heart tissues with myocardial infarction (MI) from human and mouse [135]. Moreover, targets of miR-149 include genes of the inflammatory response [133]. For example, it has been reported that miR-149 targets the adaptor proteins MyD88 and negatively regulates the cytokine production stimulated by toll-like receptors [136]. Another SNP in miR-149 gene is the rs71428439 which was also been reported to be associated with MI [134], but Liu et al. reported that rs71428439 is not associated with the development of CVD [100].

### 3.7. MIRLET7 Family of microRNA

The Let-7 family of microRNA has been implicated in the maturation of metabolism of the cardiomyocytes [137]. It has been reported that the Let-7 family of microRNA is implicated in maturation, cell size and force contractibility of human embryonic stem cells [137]. It has been suggested that maturation of the cardiomyocytes induced by the Let-7 family resulted from the promotion of fatty acids metabolism and down-regulation of the insulin signaling pathway (phosphoinositide 3 kinase (PI3K)/AKT protein kinase) [137]. Therefore, gene variations in the Let-7 family represent plausible candidates for studying the functions of the Let-7 in cardiovascular disease (Table 2).

## 4. Conclusions and Future Perspective

MiRNAs are small RNA molecules that regulate the different vital cellular process. They silence the gene expression via efficient binding to their target mRNAs. Gene polymorphisms either in the miRNA target site or in the miRNAs can disrupt this binding which can contribute to the development of diseases such as cancer, diabetes and cardiovascular disease. For example, miR-124a rs531564, miR-146a rs2910164, miR-27a rs895819 and miR-34a rs72631823 have been associated with diabetes. Whereas, the miR-196a2 rs11614913, miR-499 rs3746444 and pre-miR-27a rs895819 have been associated with CVD. In order to study the role of miRNA gene polymorphisms in the development of diabetes, one may investigate the SNPs in miRNAs that are involved in pancreatic beta cells’ proliferation (miR-375, miR-181a, miR-17, miR-24, miR-29a), survival and apoptosis (miR-577 and 200a/b/c, miR-34a) [140]. One may also consider the miRNAs which regulate the gene of elements of the insulin signaling pathway, for instance, the insulin receptor (MIR15b and MIR195), phosphoinositide 3-kinase (MIR320 and MIR378), and AKT (MIR143, and MIR106b) [140], or to investigate SNPs in miRNAs (e.g., microRNA147 [141]) that regulate genes important for an antidiabetes drug transporter such as SLC22A3 [142].

To study the roles of miRNA polymorphisms in the development of CVD, one may investigate the SNPs of the miRNAs that are involved in cardiac development [49] endothelial cell functions [50], and inflammatory response [143].

The result of GWASs have increased our understanding of drug metabolism, for instance, the SNPs that influence the warfarin metabolism have been identified and genetic testing for warfarin dosing can be performed in the clinic [144]. Moreover, GWASs revealed disease susceptibility loci that may help in identification, stratification and prognostication of individuals at disease risk [145,146,147]. There are still challenges facing the translation of genetic data to clinical practice in order to improve the prediction of disease risk loci. For instance, modern sequencing and statistical methods should be used in studies of gene–environment interactions with large cohorts to identify and characterize new SNPs relevant to T2DM prediction [146], and CVD. Most of the miRNA polymorphism studies have been conducted in Asian populations (Table 1 and Table 2). These results should await clinical validation and detailed mechanistic exploration.

The miRNAs regulate many cellular processes, and there have been more than 2000 miRNAs discovered thus far. Therefore, the role of miRNA polymorphisms (if validated) may have a bright future in the fields of pharmacogenomics, molecular epidemiology, and personalized medicine.

## Figures and Tables

**Table 1:** MicroRNA gene polymorphisms that have been associated with diabetes in different populations.

miRNA	Rs Number	Disease	No of Control/Patients	Population	Ref
1-MiR-124a	rs531564	T2DM	610/738	Chinese	[54,55]
		T2DM	185/163	Italian	
2-MiR-146a	rs2910164	T2DM	610/738	Chinese	[54,65,67]
		T1DM (DN) and T2DM (DME)	−/2948	Caucasian	
		T2DM (DPN)	−/132	Italian	
3-MiR-27a	rs895819	T2DM	185/163	Italian	[55,71,77]
		T2DM	209/204	Iranian	
		T2DM	967/995	Chinese Han	
4-MiR-34a	rs72631823	T2DM	-	INS-1, MIN6 and Hela cell lines	[78]
5-MIRLET7A2	rs1143770	T2DM/DN	62/212	Chinese	[82]
6-MiR128a	rs11888095	DPN	−/132	Italian	[67]

Abbreviations: T1DM: Type 1 diabetes mellitus, T2DM: Type 2 diabetes mellitus, DN: diabetic nephropathy, DPN: diabetic polyneuropathy.

**Table 2:** Micro-RNA gene polymorphisms that have been associated with cardiovascular diseases in different populations.

MiRNA	rs Number	Disease	No. of Control/Patients	Population	Ref
1-MiR-196a	rs11614913	CVD	834/920	Polish (With T2DM)	[37]
2-MiR-499	rs3746444	CAD	−/1004	Chinese	[105,107,115,138]
		IS	Meta-analysis	Chinese	
		CAD	480/435	Chinese	
		MI	889/919	Chinese	
3-MiR-4513	rs2168518	CAD	−/1004	Chinese	[105]
4-Pre-MiR-27a	rs895819	MI	287/646	Chinese Han population	[5]
5-Pre-MiR-146a	rs2910164	ACS	721/722	Chinese	[100,124,126,139]
		CAD	535/522	Korean	
		CAD	300/300	Iranian	
6-MiR-149	rs2292832	CAD	535/522	Korean	[126]

Abbreviations: T2DM: Type 2 diabetic mellitus, CVD: cardiovascular disease, CAD: coronary artery disease, IS: ischemic stroke, MI: myocardial infarction, ACS, acute coronary syndrome.
